# The p53 Tumor Suppressor in the Control of Metabolism and Ferroptosis

**DOI:** 10.3389/fendo.2018.00124

**Published:** 2018-04-11

**Authors:** Keerthana Gnanapradeepan, Subhasree Basu, Thibaut Barnoud, Anna Budina-Kolomets, Che-Pei Kung, Maureen E. Murphy

**Affiliations:** ^1^Program in Molecular and Cellular Oncogenesis, The Wistar Institute, Philadelphia, PA, United States; ^2^Graduate Group in Biochemistry and Molecular Biophysics, The Perelman School of Medicine, The University of Pennsylvania, Philadelphia, PA, United States; ^3^Department of Internal Medicine, School of Medicine, Washington University in St. Louis, St Louis, MO, United States

**Keywords:** p53, metabolism, ferroptosis, apoptosis, tumor suppressor

## Abstract

The p53 tumor suppressor continues to be distinguished as the most frequently mutated gene in human cancer. It is widely believed that the ability of p53 to induce senescence and programmed cell death underlies the tumor suppressor functions of p53. However, p53 has a number of other functions that recent data strongly implicate in tumor suppression, particularly with regard to the control of metabolism and ferroptosis (iron- and lipid-peroxide-mediated cell death) by p53. As reviewed here, the roles of p53 in the control of metabolism and ferroptosis are complex. Wild-type (WT) p53 negatively regulates lipid synthesis and glycolysis in normal and tumor cells, and positively regulates oxidative phosphorylation and lipid catabolism. Mutant p53 in tumor cells does the converse, positively regulating lipid synthesis and glycolysis. The role of p53 in ferroptosis is even more complex: in normal tissues, WT p53 appears to positively regulate ferroptosis, and this pathway appears to play a role in the ability of basal, unstressed p53 to suppress tumor initiation and development. In tumors, other regulators of ferroptosis supersede p53’s role, and WT p53 appears to play a limited role; instead, mutant p53 sensitizes tumor cells to ferroptosis. By clearly elucidating the roles of WT and mutant p53 in metabolism and ferroptosis, and establishing these roles in tumor suppression, emerging research promises to yield new therapeutic avenues for cancer and metabolic diseases.

## Introduction

The tumor suppressor gene *TP53* has been the most heavily studied human gene since its discovery nearly 40 years ago (1). The main reason behind this status is the critical role p53 plays in preventing cancer development, and it is widely regarded as the “guardian of the genome.” For some time it has been generally believed that p53’s role in tumor suppression is by virtue of its ability to induce the apoptosis, cell cycle arrest, and senescence of pre-cancerous cells (2). However, it is now increasingly clear that p53 regulates many other pathways in the cell and that these other pathways also play roles in p53’s ability to function as a tumor suppressor (3). In particular, p53’s role in the regulation of genes involved in metabolism and ferroptosis has been implicated in its ability to suppress tumor development. Ferroptosis is a novel cell death pathway first characterized in 2012 and can be best described as an iron-dependent, caspase-independent form of cell death driven by the formation of lipid peroxidation (4). Specifically, two mouse models containing engineered mutations in p53 that eliminate the ability of p53 to induce apoptosis and senescence both retain the ability to suppress spontaneous tumor development; both of these mutants retain the ability to transactivate genes in metabolism and ferroptosis (5, 6). A summary of the data implicating p53 in the regulation of metabolism and ferroptosis is detailed below.

## Wild-Type (WT) p53 Positively Regulates Oxidative Phosphorylation and Suppresses Glucose Metabolism

**Figure 1:**
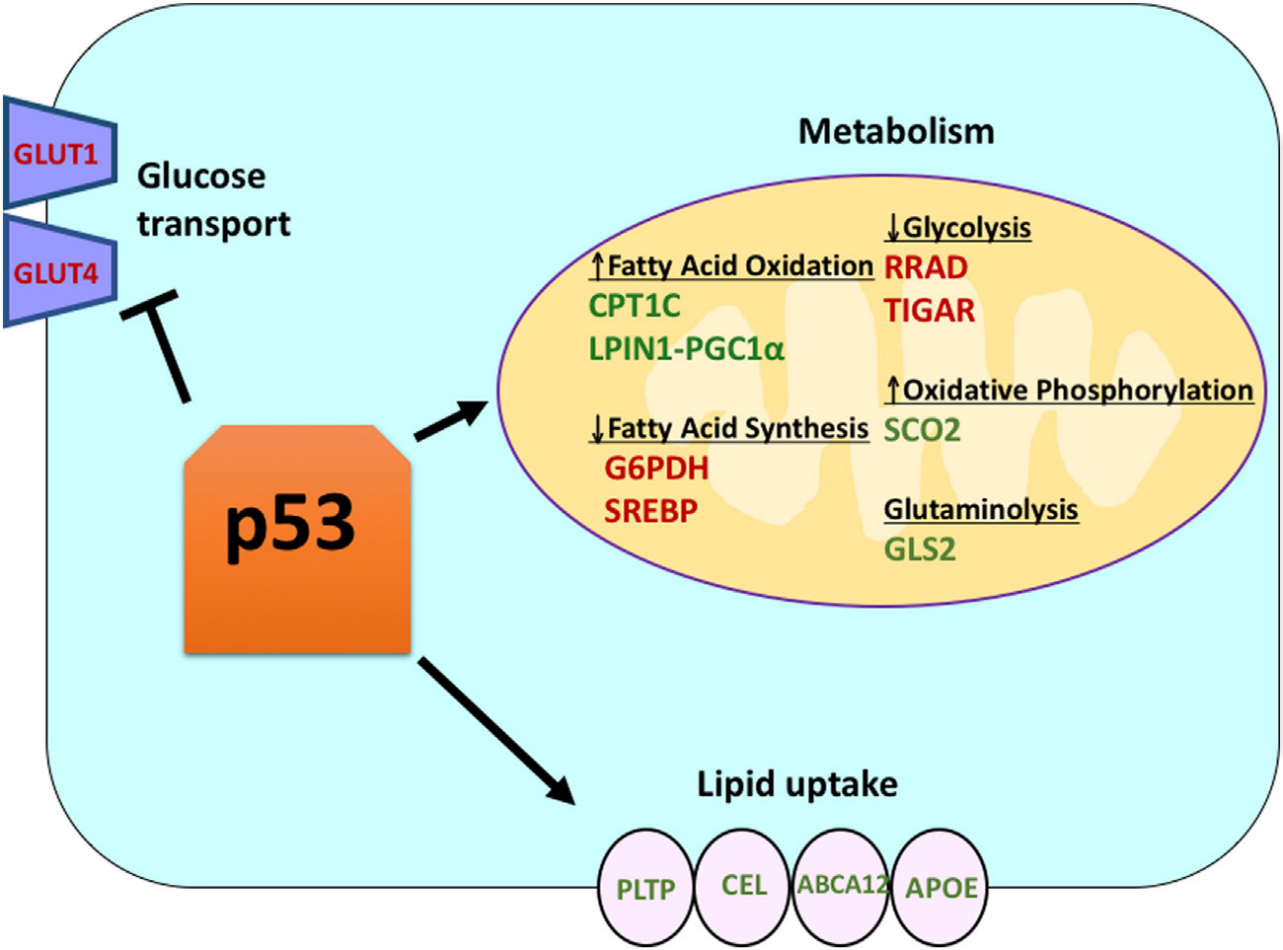
The role of wild-type (WT) p53 in metabolism. Genes positively regulated by p53 are shown in green, and genes negatively regulated by p53 are shown in red. p53 inhibits glucose transport, glycolysis, and fatty acid synthesis while it promotes lipid uptake, fatty acid oxidation, oxidative phosphorylation, and glutaminolysis.

Wild-type p53 regulates the metabolic versatility of cells by favoring mitochondrial respiration over glycolysis, in part *via* the transactivation of *SCO2* (cytochrome *c* oxidase assembly), which plays a direct role in oxidative phosphorylation (7). p53 also directly regulates the transactivation of *GLS2* (Glutaminase 2); this enzyme allows glutamine usage as an energy source for the mitochondria (8). In addition, WT p53 negatively regulates glycolysis by transcriptionally repressing the glucose transporters *GLUT1* and *GLUT4*, and by transactivating *RRAD* and *TIGAR*; both are inhibitors of glycolysis (9–11). Finally, p53 also directly binds and inhibits the enzyme glucose-6-phosphate dehydrogenase, thus suppressing glucose metabolism (12). It is clear from these and other studies that in normal, unstressed organisms, p53 directly regulates the metabolic state in a cell (Figure 1). Not surprisingly, this gene and many of its regulators are implicated in metabolic diseases, including obesity and diabetes (13).

## Mutant p53 Positively Regulates Warburg Metabolism (Aerobic Glycolysis)

In contrast to the function of WT p53, mutant p53 in tumor cells favors aerobic glycolysis, in part by enhancing the trafficking of the glucose transporter GLUT1 to the plasma membrane, hence increasing glucose import (14, 15). Following the mutation of p53, the reduced levels of *SCO2* and *GLS2* and the increased levels of *GLUT1* and *GLUT4* favor aerobic glycolysis over oxidative phosphorylation. In this manner, mutant p53 is believed to contribute to the propensity of tumor cells to utilize aerobic glycolysis in favor of oxidative phosphorylation, or so-called Warburg metabolism (15). One of the hallmarks of cancer is deregulated metabolism, generally demonstrated by this switch from aerobic glycolysis to oxidative phosphorylation. Though this results in a lower and less efficient ATP yield, it is believed that cancer cells benefit by diverting glycolytic intermediates to biosynthetic pathways necessary for rapid cell division (16). This metabolic switch also leads to decreased mitochondria-mediated apoptosis and more efficient signaling through available metabolites in cancer cells (17).

## A Common Genetic Variant in TP53 Influences its Function in Metabolism

There is a common coding region polymorphism of p53 at codon 72, encoding for either proline (P72) or arginine (R72). This amino acid variation can impact p53 function with regard to cell fate after stress. In response to DNA damage, the P72 variant of p53 predominantly triggers cell cycle arrest, while the R72 variant predominantly induces cell death, or apoptosis (18, 19). Despite these differences in function, the codon 72 variation has not been consistently associated with cancer susceptibility (20). By contrast, in human studies this polymorphism is significantly associated with increased body mass index and risk for diabetes (21, 22). This premise is supported by studies in mice, where a mouse model for these codon 72 variants shows increased high-fat diet-induced diabetes in mice with the R72 variant, compared to P72. In these studies, the p53 target genes *TNF*α and *NPC1L1* were identified as critical regulators in the increase in diet-induced obesity in R72 mice (23). Interestingly, the R72 variant has also been shown to confer increased survival of cells in response to nutrient deprivation (24). These findings have led to the hypothesis that the R72 variant of p53 arose and was selected for as populations migrated north, where cold weather would require increased fat accumulation, but where survival in response to nutrient deprivation would also be under selection (24).

## p53 Regulates Lipid Metabolism

Though p53 is well known for regulating glycolysis and the citric acid cycle, p53 also has been shown to play a role in regulating lipid metabolism (25). It is believed that WT p53 enhances fatty acid oxidation while inhibiting fatty acid synthesis, thus acting as a negative regulator of lipid synthesis (25). There are several p53 target genes with roles in lipid metabolism. Sanchez-Macedo and colleagues demonstrated that carnitine palmitoyltransferase 1C (CPT1C) is transcriptionally regulated by p53; this enzyme aids in the transport of activated fatty acids to the mitochondria. In support of a role for this p53-regulated gene in cancer, this group showed that *Cpt1c*-deficient mice display delayed tumor development and higher survival rates (26). Lipin 1 (*LPIN1*) is another p53 target gene; LPIN1 is necessary for proper adipocyte development and is induced under low nutrient conditions (27). Finck and colleagues showed that LPIN1 interacts with PGC-1α, another known p53 target gene with a role in metabolism, and that this interaction activates the expression of genes involved in promoting fatty acid oxidation (28).

In addition to directly regulating the transcription of genes involved in lipid metabolism, p53 can also regulate lipid metabolism in a manner involving direct protein–protein interaction. For example, glucose-6-phosphate dehydrogenase, which is the rate-limiting enzyme in the pentose phosphate pathway, binds to and is directly inhibited by p53, resulting in decreased NADPH production and consequently decreased fatty acid synthesis (12). The sterol regulatory element-binding proteins (SREBP) family of transcription factors modulate the expression of genes involved in cholesterol, fatty acid, triacylglycerol, and phospholipid synthesis (29–31). WT p53 represses SREBP function (32), while mutant forms of p53 bind directly to SREBP and enhance their transcriptional function, leading to increased SREBP activity in human tumors (33, 34). Consequently, mutant p53 is correlated with higher expression of sterol biosynthesis genes in human breast tumors (34, 35). Finally, AMP-activated protein kinase (AMPK) is an enzyme that is activated under low nutrient levels or energy stress and is known to inhibit fatty acid synthesis by interacting with acetyl-CoA-carboxylase and SREBP-1 (36, 37). Zhou and colleagues demonstrated that mutant p53 preferentially binds to and inhibits AMPK, leading to increased fatty acid synthesis. As a result, mutant p53 proteins lead to increased AMPK signaling, contributing to invasive cell growth of tumor cells (33). A lesser explored area is the role of p53 in lipid transport. It has been shown that p53 transcriptionally regulates apolipoprotein B (apoB) and apoB editing enzyme complex 1, indicating the role of p53 in regulating atherogenic lipoproteins (38). Microarray analysis of human liver-derived cells identified phospholipid transfer protein, ATP binding cassette A12, and carboxyl ester lipase as three p53 target genes that all play a role in lipid transport (39, 40). Overall, though it is clear that p53 plays a key role in mediating lipid synthesis and metabolism, the contribution of this pathway, and these p53 target genes, to tumor suppression by p53 remains to be determined (Figure 1).

## Ferroptosis is a Novel Cell Death Pathway Driven by Lipid Peroxidation

In 2012, Dixon and colleagues discovered a novel form of regulated cell death called ferroptosis. Ferroptosis is an iron-dependent, caspase-independent form of cell death resulting from the accumulation of oxidized lipids (4, 41). This process is driven by the inactivation of glutathione peroxidase 4 (GPX4), an enzyme that is responsible for converting lethal lipid hydroperoxides to non-toxic lipid alcohols, which requires glutathione in order to function (41). It is believed that peroxidation of polyunsaturated fatty acids (PUFAs) is the driving impetus for cell death by ferroptosis. PUFAs contain bis-allylic protons that can easily be abstracted and produce radicals that will react with oxygen, creating more radicals and resulting in a chain reaction of lipid reactive oxygen species (42). The exact mechanism of cell death by ferroptosis remains unknown, but one hypothesis is that the lipid damage leads to the destruction of the plasma membrane (43). It has been speculated that ferroptosis could be a mechanism of tumor suppression that works by eliminating cells that are nutrient deprived or have been exposed to an environmental stress or infection.

## Pharmacologic Regulation of Ferroptosis

Ferroptosis can be induced using inhibitors of system ${\text{x}}_{\text{c}}^{-}$ such as erastin, or analogs such as glutamate and sorafenib, which inhibit the import of cystine, resulting in depleted glutathione and subsequent inactivation of GPX4. Alternatively, ferroptosis can be induced by (1S,3R)-RSL3 (hereafter referred to as RSL3), which directly binds to and inhibits GPX4 (4, 5, 42). Buthione sulfoximine, FIN56, FINO2, CCl_4_, and cisplatin are other agents that have been demonstrated to induce ferroptosis in cells. Death by ferroptosis can be prevented by suppressing lipid peroxidation, which can be accomplished by using lipophilic antioxidants, such as ferrostatin-1, liproxstatin-1, or vitamin E. Iron chelators such as deferoxamine or cicloprox are another tool used to suppress ferroptosis by reducing the levels of iron. Depleting PUFAs or adding monounsaturated fatty acids to cell culture media can also rescue cells from ferroptosis (42, 44).

## Ferroptosis is Implicated in p53-Mediated Tumor Suppression

In 2012, Gu and colleagues developed a mouse model in which three normally acetylated lysine residues in the DNA-binding domain of p53 were mutated to arginine, and therefore could not be acetylated; this mouse is referred to as the 3KR mouse. Notably, cells from the 3KR mouse are unable to undergo p53-dependent apoptosis, cell cycle arrest, or senescence, and indeed the 3KR mutant of p53 fails to transactivate the majority of p53 target genes. Interestingly, this mouse model does not spontaneously develop cancer, implying that p53 could suppress tumor development independent of senescence or apoptosis (45). This group found that the mutant 3KR protein retains the ability to undergo ferroptosis and regulate cystine metabolism by regulating the expression of the cystine importer *SLC7A11*; this suggested that ferroptosis might be one pathway that underlies p53-mediated tumor suppression. When wild type and 3KR MEFs were treated with the ferroptosis inducer Erastin, almost 50% cell death was observed whereas p53 null MEFs exhibited 20% cell death; this indicates that p53 sensitizes cells to ferroptosis, and also that other key regulators also play a role in ferroptosis (5). Subsequently, Gu and colleagues identified an additional acetylation site at lysine 98 of p53, and they generated a mouse model in which all four acetylation sites were mutated to arginine (4KR). Interestingly, the 4KR mutant was unable to regulate genes involved in ferroptosis like *SLC7A11*, and unlike the 3KR mutant was unable to suppress tumor development (46). Though at present correlative, these data implicate the role of p53 in ferroptosis in its ability to suppress tumor development.

## In Non-Transformed Cells, p53 Positively Regulates Ferroptosis

In addition to *SLC7A11*, several other direct p53 target genes have been discovered to play a role in ferroptosis. These include *GLS2, PTGS2*, and *SAT1*. Studies from two separate groups support the role of *GLS2* in ferroptosis, which is known to decrease glutathione and increase cellular ROS levels. Jiang and colleagues used ferroptosis inhibitors combined with glutaminolysis inhibitors to inhibit Erastin-induced ferroptosis, thereby demonstrating that ferroptosis requires glutaminolysis and *GLS2* (47). Murphy and colleagues showed that a polymorphic variant of p53 was able to induce growth arrest and senescence in both human and murine cells but failed to repress *SLC7A11* or transactivate *GLS2*. This variant was markedly impaired at inducing ferroptosis and suppressing tumor development, thus again implicating the role of p53 in ferroptosis-mediated tumor suppression (48). Another p53 target gene with a role in ferroptosis is *PTGS2*, a gene encoding the enzyme cyclooxygenase-2. Stockwell and colleagues first showed that the induction of ferroptosis using Erastin and RSL3 led to the upregulation of *PTGS2* (41). Notably, *PTGS2* was not upregulated by ferroptosis inducers in p53-null cells, suggesting that this regulation is p53 dependent (5). Presently, the upregulation of *PTGS2* is widely used as a ferroptosis marker (5, 41).

**Figure 2:**
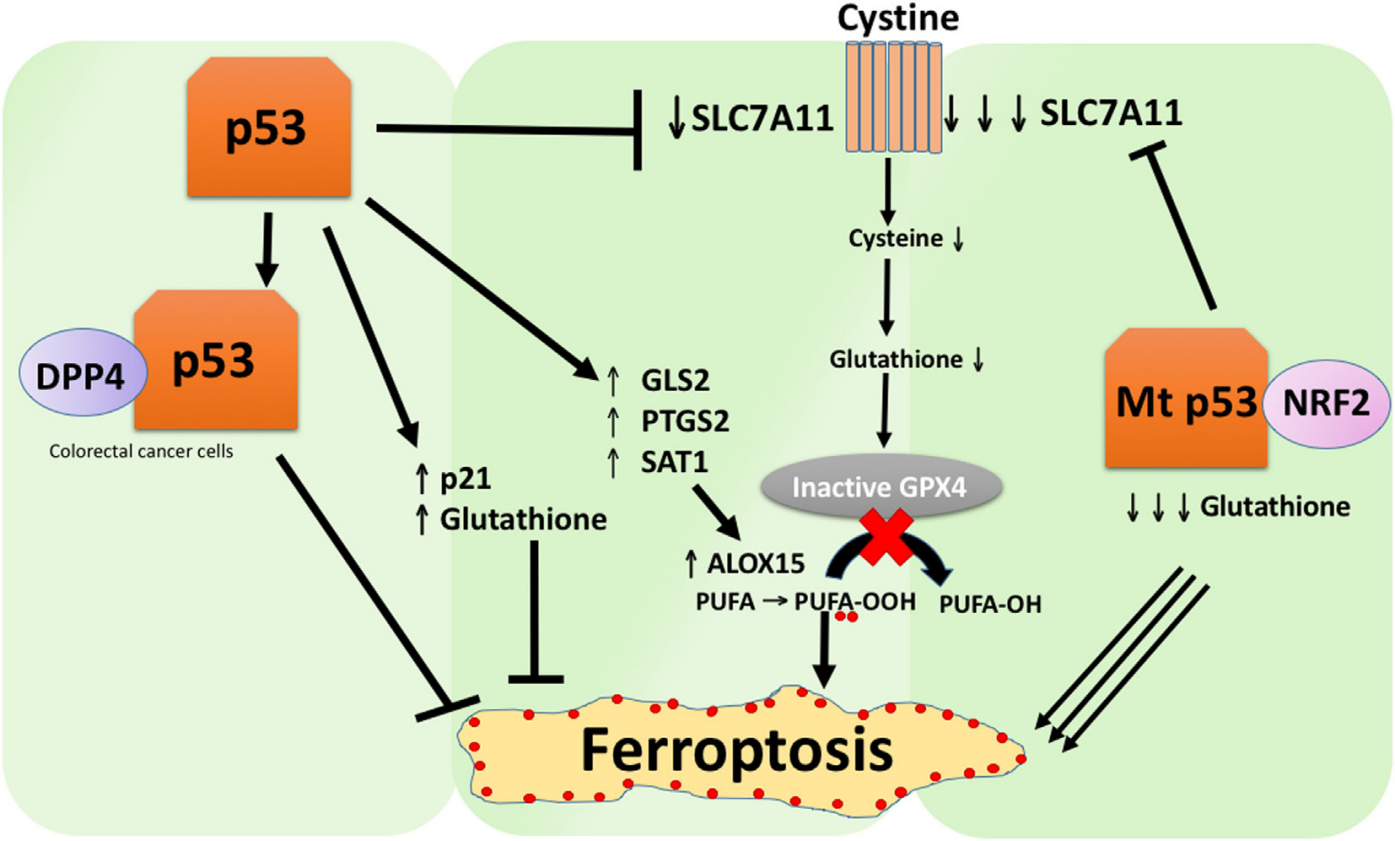
The various roles of p53 in ferroptosis. Inhibition of glutathione peroxidase 4 (GPX4), the key enzyme that catalyzes the conversion of polyunsaturated fatty acids (PUFAs) containing peroxides to alcohols, is the key driver of ferroptosis. Depending on the context, p53 can suppress ferroptosis (such as in colorectal cancer cells) or promote ferroptosis. Mutant p53 sensitizes cells to ferroptosis even more than wild-type p53.

A recent study by the Gu group showed that the p53 target gene *SAT1* regulates ferroptosis (49). The authors identified *SAT1* as a direct target of p53 and showed that silencing of *SAT1* reduced cell death induced by reactive oxygen species in cells with WT p53, but had no effect in p53-null cells. Mechanistically, this group showed that *SAT1* increases the level and activity of arachidonate 15-lipoxygenase, an iron-binding enzyme that oxidizes PUFAs and increases lipid peroxidation. Notably, this study showed that neither p53 nor SAT1 alone appear to be sufficient to induce ferroptosis. Instead, the combined data are more consistent with the premise that p53, by virtue of regulating genes that contribute to ferroptosis, regulates the sensitivity of cells to this pathway, rather than directly induces ferroptosis. Whether p53 regulates other genes involved in ferroptosis remains to be determined (Figure 2).

## In Some Cells, p53 Negatively Regulates Ferroptosis

A study recently published by Tarangelo and colleagues shows that p53 negatively regulates ferroptosis in cancer cells (50). This group found that pre-treating cells with Nutlin-3, a compound that stabilizes p53 delays the onset of ferroptosis in several cell types. The delayed onset of ferroptosis was found to depend on *CDKN1A* (encoding p21), a critical p53 transcriptional target. The mechanism through which p21 delays ferroptosis has yet to be elucidated, but it is believed that the conservation of intracellular glutathione may be a contributing factor for reduced ferroptosis sensitivity. The authors conclude that the p53–p21 axis enables cancer cells to survive under conditions of metabolic stress, such as cystine deprivation, by suppressing the onset of ferroptosis (50). A recent study showed that p53 inhibits ferroptosis in colorectal cancer cells by binding to the enzyme dipeptidyl-peptidase-4 (DPP4), which is a modulator of ferroptosis and lipid metabolism. Mechanistically, this study showed that p53 antagonizes ferroptosis by sequestering DPP4 in a nuclear enzymatic inactive pool. In the absence of p53, DPP4 is free to interact with and form a complex with NOX1; this leads to increased lipid peroxidation and ferroptosis. Inhibition of DPP4 suppresses ferroptosis significantly, whereas overexpression of DPP4 triggers Erastin sensitivity, particularly in p53-depleted cells (51). The bidirectional control of ferroptosis by p53 through transcription-dependent and transcription-independent mechanisms may be context or cell-type dependent (Figure 2).

## The P47S Polymorphism of TP53 Affects Ferroptosis and Tumor Suppression

In addition to missense mutations, there are several functionally significant single-nucleotide polymorphisms (SNPs) in the TP53 gene and other proteins known to regulate this pathway (such as *MDM2* and *MDM4*). The Pro47Ser variant (hereafter S47) is the second most common SNP found in the p53 coding region (after Pro72Arg) that alters the amino acid sequence of the protein. To better elucidate the impact of this variant on p53 function and cancer risk, the Murphy group generated a humanized p53 knock-in mouse model, in which exons 4–9 of murine p53 were replaced by human p53 exons containing either the wild type or the S47 variant (52–55). The majority of S47 mice spontaneously developed tumors of various histologic types, particularly liver cancer, between 12 and 18 months of age, unlike WT p53 mice (48). In mouse embryonic fibroblasts and human lymphoblastoid cell lines, the S47 variant showed impaired programmed cell death in response to cisplatin and other genotoxic stresses. Mechanistically, the S47 variant is defective for transactivation of genes involved in metabolism, such as *Gls2* (glutaminase 2) and *Sco2* (48). Consistent with the role of *Gls2* in ferroptosis, this group found that S47 cells were markedly resistant to the ferroptosis-inducing agents Erastin and RSL3 (47, 48). This defect may contribute to the tumor-prone phenotype observed in S47 mice.

## Mutant p53 Sensitizes Tumor Cells to Ferroptosis

Wild-type p53 negatively regulates the expression of the cystine importer *SLC7A11*, which inhibits sensitivity to ferroptosis (5). Although this regulation occurs in normal cells, in tumor cells, other mediators of *SLC7A11* appear to predominate in the regulation of this gene. For example, the master antioxidant transcription factor NRF2 can also regulate the expression of *SLC7A11* at the transcriptional level, and NRF2 has been implicated as a key player in protecting cancer cells against ferroptosis. For example, inhibition of NRF2 in hepatocellular cancer cells increases the anti-cancer activity of Erastin and Sorafenib *in vivo* (56). Mutant forms of p53 can inhibit NRF2 function by direct interaction, and one group found that tumors with mutant p53 contain very low levels of *SLC7A11*, and thus show increased sensitivity to ferroptosis. Notably, overexpression of *SLC7A11* in mutant p53 models led to drug resistance, suggesting that levels of *SLC7A11* expression must be considered when targeting mutant p53 driven cancers with ferroptosis-inducing compounds (57). In support of this premise, recent work in colorectal (CRC) cancer, where mutation or deletion of p53 is a frequent event, showed that human CRC cell lines harboring mutant p53 were far more sensitive to Erastin-mediated cell death when compared to CRC cells with WT p53. To validate these findings, they showed that knock in of a p53 hotspot mutation in both HCT116 and SW48 cells restored sensitivity to Erastin (51). These data highlight a novel mechanism by which cancers driven by mutant p53 can be exploited using targeted therapy.

## Conclusion

The role of p53 in metabolism is quite clear and possibly even intuitively obvious: WT p53 limits glucose metabolism and lipid synthesis, while mutant p53 appears to do the opposite. The contribution of its metabolic role to tumor suppression by p53, and to the ability of mutant p53 to drive tumor progression, remains to be unequivocally proven. The role of p53 in the regulation of ferroptosis, and the contribution of this function, to tumor suppression is even less clear. While compelling data from mouse models supports the premise that p53 regulates the sensitivity of cells to ferroptosis, this may be restricted to the ability of basal p53 to suppress spontaneous tumor development, and in oncogene-stressed mouse models, it is clear that senescence and apoptosis play the predominant role. Similarly, p53 may regulate ferroptosis sensitivity in a cell type-specific manner. More studies in animal models, with attention to ferroptosis in different tissues, need to be done to more fully understand the role of p53 in ferroptosis and ferroptosis in tumor suppression. Additionally, a clearer idea of what p53-target genes play a role in sensitivity to ferroptosis needs to be attained. Resolution of these questions should provide for much needed novel avenues to combat tumors with mutant p53.

## Author Contributions

KG, SB, TB, AB-K, C-PK, and MM each wrote one to two paragraphs of this article. KG and SB did the figure. KG and MM outlined the chapter.

## Conflict of Interest Statement

The authors declare that the research was conducted in the absence of any commercial or financial relationships that could be construed as a potential conflict of interest. The reviewer OAF and handling Editor declared their shared affiliation.
